# Astatine-211-Labeled Gold Nanoparticles for Targeted Alpha-Particle Therapy via Intravenous Injection

**DOI:** 10.3390/pharmaceutics14122705

**Published:** 2022-12-02

**Authors:** Xuhao Huang, Kazuko Kaneda-Nakashima, Yuichiro Kadonaga, Kazuya Kabayama, Atsushi Shimoyama, Kazuhiro Ooe, Hiroki Kato, Atsushi Toyoshima, Atsushi Shinohara, Hiromitsu Haba, Yang Wang, Koichi Fukase

**Affiliations:** 1 Department of Chemistry, Graduate School of Science, Osaka University, 1-1 Machikaneyama, Toyonaka 560-0043, Osaka, Japan; 2 Division of Science, Institute for Radiation Sciences, Osaka University, 1-1 Machikaneyama, Toyonaka 560-0043, Osaka, Japan; 3 Core for Medicine and Science Collaborative Research and Education, Forefront Research Center, Graduate School of Science, Osaka University, 1-1 Machikaneyama, Toyonaka 560-0043, Osaka, Japan; 4 Department of Nuclear Medicine and Tracer Kinetics, Osaka University Graduate School of Medicine, 2-2 Yamadaoka, Suita 565-0871, Osaka, Japan; 5 Faculty of Health Science, Osaka Aoyama University, 2-11-1 Niina, Minoh 562-8580, Osaka, Japan; 6 Nishina Center for Accelerator-Based Science, RIKEN, 2-1 Hirosawa, Wako 351-0198, Saitama, Japan

**Keywords:** astatine-211, gold nanoparticles, targeted alpha-particle therapy, cancer therapy, pancreatic cancer, intravenous administration

## Abstract

Alpha-particle radiotherapy has gained considerable attention owing to its potent anti-cancer effect. ^211^At, with a relatively short half-life of 7.2 h, emits an alpha particle within a few cell diameters with high kinetic energy, which damages cancer cells with high biological effectiveness. In this study, we investigated the intravenous injection of ^211^At-labeled gold nanoparticles (AuNPs) for targeted alpha-particle therapy (TAT). Different kinds of surface-modified gold nanoparticles can be labeled with ^211^At in high radiochemical yield in 5 min, and no purification is necessary. The in vivo biodistribution results showed the accumulation of 5 nm ^211^At-AuNPs@mPEG at 2.25% injection dose per gram (% ID/g) in tumors within 3 h via the enhanced permeability and retention (EPR) effect. Additionally, we observed a long retention time in tumor tissues within 24 h. This is the first study to demonstrate the anti-tumor efficacy of 5 nm ^211^At-AuNPs@mPEG that can significantly suppress tumor growth in a pancreatic cancer model via intravenous administration. AuNPs are satisfactory carriers for ^211^At delivery, due to simple and efficient synthesis processes and high stability. The intravenous administration of 5 nm ^211^At-AuNPs@mPEG has a significant anti-tumor effect. This study provides a new framework for designing nanoparticles suitable for targeted alpha-particle therapy via intravenous injection.

## 1. Introduction

Radionuclide cancer therapeutics are pharmaceuticals that use short-lived radionuclides that emit α or β particles to kill cancer cells via intracellular or intratumoral radiation. Recently, much attention has been given to the potent cancer therapeutic efficacy of targeted alpha-particle therapy (TAT) (radionuclides that emit α particles) [1,2]. Compared to β particles, α particles possess higher kinetic energy and ionization power. Therefore, TAT can significantly induce DNA double-strand breaks (DSB) that effectively damages cancer cells, while damage to normal cells is limited owing to the short penetration range. Considering the decay process, half-life, and manufacturing processes of radionuclides, there are a limited number of α-emitting radionuclides suitable for TAT, such as ^223^Ra, ^225^Ac, and ^211^At [2]. So far, ^223^Ra dichloride is the only TAT drug approved by the US Food and Drug Administration and European Medicines Agency for treating patients with castration-resistant prostate cancer and symptomatic bone metastases [3]. ^223^Ra mimics calcium and forms complexes with hydroxyapatite in areas with increased bone turnover. Thus, the selective accumulation of radio-pharmaceuticals is vital for TAT to effectively suppress adverse effects on normal cells. Generally, TAT drugs are delivered to cancer cells by binding radionuclides to carrier (vehicle) drugs that target cancers. Radionuclide ^225^Ac, which has been extensively studied for cancer treatments, can be attached to molecular targeted drugs via chelate formation. For instance, ^225^Ac-PSMA-617 was of particular interest owing to its therapeutic effect on tumors that metastasized throughout the body. After ^225^Ac-PSMA-617 treatment, the majority of the tumor seemed to disappear, and prostate-specific antigen tests further indicated that metastasized prostate cancer was cured to a large extent [4]. Additionally, a study at Osaka University also showed that ^225^Ac-labeled fibroblast activation protein inhibitors (FAPI) could be used to treat fibroblast activation protein (FAP)-expressing pancreatic cancer [5]. Globally, especially in Japan, another kind of α particle-emitting radionuclide, astatine-211 (^211^At), has attracted considerable attention and multiple ^211^At-labeled compounds are currently under development [6,7].

Astatine is one of the elements in halogens; thus, it shares some chemical properties with iodine. Sodium-iodide symporter plays a significant role in the accumulation of iodine as well as astatine [8]. Taking advantage of this property, a research team led by Osaka University used sodium astatide (^211^At-NaAt) to suppress refractory thyroid cancer [9]. Since astatide was found to be readily oxidized to other chemical species with higher oxidative states, the addition of ascorbic acid to the ^211^At solution can significantly increase the radiochemical purity of astatide and enhances uptake by the thyroid in rats. Furthermore, in a murine xenograft model, the mice subjected to 1 MBq of ^211^At-NaAt survived nearly three times as long as untreated mice, and their tumors were several-fold times smaller. Currently, clinical research on cancer treatment using ^211^At-NaAt is being actively promoted at Osaka University [10].

Unlike ^225^Ac, ^211^At can be introduced to carriers via covalent bonding. Hence, small-molecule drugs targeting cancer cells can be used as a carrier. Kaneda-Nakashima et al. in Osaka University prepared ^211^At-labeled α-methyl-L-tyrosine (^211^At-AAMT) targeting a cancer-specific L-type amino acid transporter 1 (LAT1), which is highly expressed in cancer cells, but rarely expressed in normal cells [11]. ^211^At-AAMT exhibited a high affinity for LAT1, inhibited tumor cells, and caused DNA DSBs in vitro. ^211^At-AAMT was selectively accumulated in tumors and had remarkable anti-cancer effects in a murine tumor model [12].

Our research explored the substantial potential of gold nanoparticles (AuNPs) as the carrier for the targeted delivery of ^211^At via intravenous administration. In a previous study, we found that ^211^At was efficiently introduced to AuNPs by mixing ^211^At with AuNPs solution for 5 min with a high radiochemical yield (RCY), and no purification process was necessary [13]. Since AuNPs facilitate a variety of surface modifications and have been widely used as drug carriers for diagnostic or therapeutic agents [14,15,16,17], we investigated the applications of ^211^At-labeled gold nanoparticles (^211^At-AuNPs) for TAT. We firstly showed that the intratumoral administration of ^211^At-AuNPs can strongly suppress tumor growth, and also showed the stability of ^211^At-AuNPs in the body [13]. Although some studies have shown the in vitro and in vivo studies of ^211^At-AuNPs [13,18,19], the anti-tumor effect of ^211^At-AuNPs via intravenous injection has not been previously investigated. Intravenous injection is one of the most commonly used drug delivery methods that can rapidly deliver drugs throughout the body. For systemic metastatic tumors, intravenous injection is an excellent option in TAT. Intravenous injection requires a high drug accumulation capacity in tumor tissue, which may otherwise result in nonspecific irradiation of normal cells and tissues.

Here, four kinds of functional AuNPs were investigated. These can be divided into two groups: nanoparticles modified with methoxy polyethyleneglycol (mPEG), which are 5 nm and 30 nm AuNPs@mPEG, and nanoparticles modified with polyhitidine (H16) peptide and mPEG, which is 5 nm AuNPs@H16, or multi-modified with cyclic arginylglycylaspartic acid (RGD) peptide, H16 peptide and mPEG, which is 5 nm AuNPs@H16/RGD. The nanoparticles modified with mPEG were expected to accumulate in tumor tissues via the enhanced permeability and retention (EPR) effect. Since the half-life of ^211^At is short, the rapid accumulation of ^211^At-labeled compounds in cancer cells has a pivotal role in alpha therapy with minimal side effects on normal tissues. We took advantage of the EPR effect and the fact that cancer cells have a higher nanoparticle uptake rate than normal cells to develop a novel TAT drug.

Additionally, we investigated the internalization and DNA DSBs induction of 5 nm ^211^At-labeled AuNPs@H16 and AuNPs@H16/RGD in vitro, and we also investigated the biodistribution of ^211^At-labeled AuNPs in vivo. The H16 peptide [20] and cyclic RGD [21,22] can target tumor pH and the integrin α_v_β_3_, respectively. Polyhistidine peptides were developed for pH-responsive tumor targeting because of the lower pH state formed in solid tumor tissues [20]. The integrin α_v_β_3_ is highly expressed in tumor cells and the new-born vessels between tumor tissue. Many human pancreatic cancer cells, including the PANC-1 cell line used in this research, were reported for their high expression of integrin α_v_β_3_ [23]. The modification of these peptides was hence expected to improve the tumor targeting and cellular uptake of AuNPs.

## 2. Materials and Methods

### 2.1. Astatine-211 Production

^211^At was produced in the ^209^Bi(α,2n)^211^At reaction using the RIKEN AVF Cyclotron and purified as previously described [9,13,24].

### 2.2. AuNPs Modification and Characterization

AuNPs (5 nm and 30 nm) (OD 1, stabilized suspension in citrate buffer) were purchased from Sigma-Aldrich (St. Louis, MO, USA). For mPEG modification, mPEG thiol (Mn 6000) was added to aqueous solutions containing AuNPs of 5 nm and 30 nm so that the final concentration of mPEG thiol was 0.1 M. The reaction mixtures were stirred at room temperature for 2 h. After that, the mPEG-modified AuNPs were purified by centrifugation as we reported in previous study in order to obtain 5 nm and 30 nm AuNPs@mPEG. H16 peptide (2-mercaptopropionic acid-HHHHH HHHHH HHHHH H-NH2 trifluoroacetate salt) and RGD peptide (cyclo(Arg-Gly-Asp-D-Phe-Lys[Cys])) were purchased from Peptide Institute. Inc. (Ibaraki, Osaka, Japan). For H16 and RGD peptide modifications, we first added the mPEG thiol (Mn 2000) to the 5 nm AuNPs solutions, and the mixtures were stirred at room temperature for 10 min. The mixtures were centrifuged once, and the solvent was changed to 0.1 M phosphate buffer (pH 6.0). Next, the H16 peptide solution or H16 and RGD peptide mixture solution was added to the mPEG-modified AuNPs dissolved in phosphate buffer (pH 6.0), and the solutions were stirred for 3 h at room temperature. After the reaction, the H16-modified AuNPs or H16 and RGD double-modified AuNPs were purified and centrifuged using Amicon^®^ ultra-0.5 centrifugal filter devices (50K) at 10,000× *g* for 5 min. Following centrifugation, 0.1 M phosphate buffer (pH 6.0) was added to the devices. This operation was performed twice to obtain 5 nm AuNPs@H16 or AuNPs@H16/RGD. AuNPs@mPEG (120 nm) was prepared as previously described [13]. The quality of the synthesized AuNPs was confirmed by transmission electron microscopy (TEM) (JEM-2100; JEOL Ltd., Tokyo, Japan). Dynamic light scattering (DLS) and zeta potential measurements of surface-modified AuNPs were measured using Zetasizer Ultra (Malvern Panalytical Ltd., Malvern, UK). The ultraviolet–visible (UV–Vis) absorption spectrum of modified AuNPs was determined using a UV–Visible/NIR spectrophotometer (V-730; JASCO Corp., Tokyo, Japan).

### 2.3. ^211^At-Labeling of AuNPs

The preparation of ^211^At solution and the ^211^At-labeling of AuNPs were carried out according to our previous method [13]. Distilled water (OTSUKA, Tokyo, Japan) was used as the solvent for the preparation of 5 nm and 30 nm ^211^At-AuNPs@mPEG, whereas 0.1 M phosphate buffer (pH 6.0) (Wako, Japan) was used as the solvent for the preparation of peptide-modified ^211^At-AuNPs. ^211^At aqueous solution (30 μL, 4.5–6.0 MBq) was added to 70 μL of modified AuNPs aqueous solution, and the mixture was shaken at room temperature for 5 min. The solution of AuNPs was diluted with distilled water (OTSUKA, Tokyo, Japan) to 300 μL and 600 μL of saline (OTSUKA, Tokyo, Japan) was added to give the sample solutions used for biological assay. For the distribution and therapeutic assays, 100 μL of the sample solution was injected into each mouse.

The radioactivity of ^211^At-AuNPs was then measured by a germanium semiconductor detector BE-2020; Mirion Technologies (Canberra), Inc., Meriden, CT, USA). Then, the solution was transferred into 50K Amicon^®^ ultra centrifugal filters and separated by centrifugal filtration (6000× *g*, 10 min) twice. Through the radioactivity measurement of reaction tubes, filtrates, filter devices and tips, we could calculate the RCY of the astatination reaction. Radioactive decay correction was conducted according to the measurement time.

### 2.4. In Vivo Biodistribution and Therapy Efficacy

The experimental protocol was approved by the Animal Care and Use Committee of the Osaka University Graduate School of Science (approval code: 2019-02-1, approval date: 1 April 2019, validity period: five years). All animals were housed under a 12 h dark–light cycle (light from 08:00 to 20:00) at 25 ± 1 °C with ad libitum food (CRF-1: Oriental Yeas Co., Ltd., Tokyo, Japan) and water in a multi-chamber animal housing System (Nippon Medical & Chemical Instruments. Co., Ltd., Osaka, Japan). BALB/c-nu/nu mice were purchased from Japan SLC Inc. (Hamamatsu, Japan). PANC-1 cells were cultured at 37 °C in Dulbecco’s modified Eagle medium (DMEM) containing 10% fetal bovine serum and 1% antibiotics in a humidified incubator with 5% CO_2_. Cultured cells were washed in phosphate-buffered saline (PBS) and harvested with trypsin.

Tumor xenograft models were established by the subcutaneous injection of 1 × 10^7^ cells in 0.2 mL of serum-free medium and Matrigel (1:1; BD Bioscience, Franklin Lakes, NJ, USA) into male BALB/c-nu/nu mice. 

For the distribution assay, PANC-1 mice (n = 32; twelve weeks old; male body weight = 24.4 ± 1.1 g) were used for evaluation at 3 h and 24 h following the administration of the 5 nm ^211^At-AuNPs@mPEG (241.5 ± 22.3 kBq/mouse), 30 nm ^211^At-AuNPs@mPEG (211.4 ± 3.4 kBq/mouse), ^211^At-AuNPs@H16 (405.7 ± 11.4 kBq/mouse), and ^211^At-AuNPs@H16 H16/RGD (410.3 ± 10.2 kBq/mouse). All organs were packed into a zippered polyethylene bag and radioactivity was measured with a gamma counter (2480 Wizard^2^, PerkinElmer, Waltham, MA, USA). 

For the therapeutic assay, PANC-1 xenograft mice (n = 12, 7 weeks old; body weight = 23.3 ± 1.1 g) were used when the tumor size reached approximately 50 mm^3^ on average. The mice were divided into three groups according to the injected dose (1 MBq (989.2 ± 6.3 kBq/mouse, n = 4), 0.5 MBq (515.9 ± 8.6 kBq/mouse, n = 4), and control (n = 4)). The control group received no ^211^At-labeled AuNPs. Tumor sizes and body weights were measured thrice per week. The mice were euthanized when the tumor size reached more than 10% of the total weight. The mice were followed for 30 days.

### 2.5. Internalization Evaluation

PANC-1, MDA-MB-231, and Hs 578Bst cells were cultured at 37 °C in D-MEM containing 10% fetal bovine serum and 1% antibiotics in a humidified incubator with 5% CO_2_. The cultured cells were washed in PBS and harvested with trypsin as previously described.

The internalization of AuNPs (no radionuclide) were evaluated using reflectance imaging as we reported in previous study [13]. Briefly, PANC-1 cells (1 × 10^4^ cells/well in 100 µL of medium), MDA-MB-231 cells (5 × 10^4^ cells/well in 100 µL of medium), and Hs 578Bst cells (5 × 10^4^ cells/well in 100 µL of medium) were seeded on to 96-well plates (glass bottom) and treated with the different kinds of AuNPs for 24 h. The cells were then washed, and fixed with 4% paraformaldehyde solution, and the cell nucleus was stained by Hoechst 33342. Micrographs were taken with a Nikon A1R + inverted confocal microscope (Nikon Corp., Tokyo, Japan) and a Plan Apo VC water-immersion objective lens (60×, NA = 1.20; Nikon Corp., Tokyo, Japan). 

The internalization of ^211^At-labeled AuNPs was quantitated by the determination of the radioactivity. Briefly, the PANC-1 cells were seeded in 96-well plates and the different kinds of AuNPs solutions were added to each well. Each sample was added to the cells and incubated for 5 h. After treatment, the cells were washed twice with 1X PBS and lysed with 1N NaOH. The radioactivity levels in the lysed cell samples were measured with a gamma counter, 2480 Wizard^2^ (PerkinElmer, Inc., Waltham, MA, USA). The protein levels were determined with a BCA protein assay kit (Thermo Fisher Scientific, Waltham, MA, USA).

The internalization of 120 nm AuNPs@mPEG in MDA-MB-231 and Hs 578Bst cell lines was investigated by reflectance imaging [13]. Images were taken using a Nikon A1R + inverted confocal microscope (Nikon Corp., Tokyo, Japan) and a Plan Apo VC water-immersion objective lens (60×, NA = 1.20; Nikon, Tokyo, Japan) (Figure S5).

### 2.6. Statistical Analysis

The results are expressed as the mean ± standard deviation. For the in vivo results, comparisons between groups were performed using unpaired *t*-tests in Microsoft Excel (version 2016). For multiple comparisons among the three groups, Bonferroni correction was performed. For the in vitro results, the *p*-value was calculated with one-way ANOVA using GraphPad Prism software. Differences were considered statistically significant at *p* < 0.05.

## 3. Results

### 3.1. ^211^At-Labeled AuNPs Synthesis

**Figure 1:**
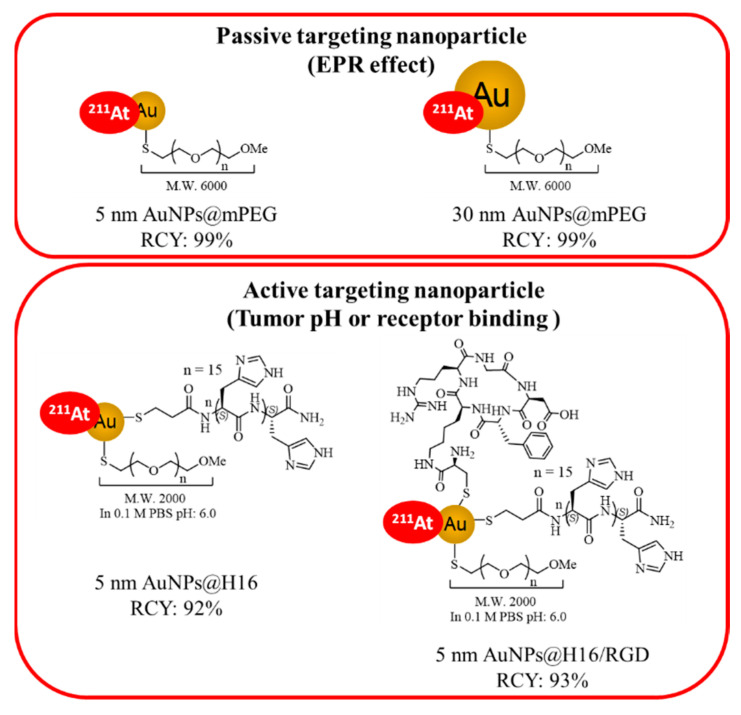
Four kinds of ^211^At-labeled functional AuNPs designed for the study: 5 nm and 30 nm ^211^At-AuNPs@mPEG; 5 nm ^211^At-AuNPs@H16 and ^211^At-AuNPs@H16/RGD. RCY: radiochemical yield.

Four kinds of ^211^At-labeled functional AuNPs (5 nm ^211^At-AuNPs@mPEG, 30 nm ^211^At-AuNPs@mPEG, 5 nm ^211^At-AuNPs@H16, and 5 nm ^211^At-AuNPs@H16/RGD) were designed as shown in Figure 1. 

The synthesized surface-modified AuNPs were evaluated via TEM, DLS, and UV–Vis, and the results are shown in Figure S1 and Table S1. All kinds of AuNPs were found to be approximately spherical and reasonably monodispersed. The different surface modifications affected their zeta-potentials. The mPEG-modified AuNPs were well dispersed in an aqueous solution. While the two kinds of peptide-modified AuNPs were aggregated in water as the solvent during the modification process, they were dispersed and stable in PB solution (pH 6.0). To evaluate the stability of 5 nm ^211^At-AuNPs@H16 and ^211^At-AuNPs@H16/RGD, the samples were diluted in phosphate buffer (pH 7.4 or 8.0), and DLS measurement was performed. The two kinds of peptide-modified AuNPs were reasonably monodispersed and no notable size change was observed (Table S2). 

After 5 min of mixing the AuNPs and ^211^At solution, the ^211^At-labeling efficiency was evaluated as previously described. The labeling conditions and RCY are shown in Table S3 and Figure S2. Although there was some adhesion to the reaction tube walls and pipette tips, the free ^211^At in the filtrate was less than 1%, and all final RCYs were above 90%.

### 3.2. Biodistribution Study

Four kinds of ^211^At-AuNPs were intravenously injected into mice, and after 3 h or 24 h, the radioactivity of organ tissues was measured to evaluate the biodistribution. The percentage distribution of injected dose per gram (% ID/g) and the percentage distribution of injected dose (% ID) of 5 nm and 30 nm ^211^At-AuNPs@mPEG is shown in Table 1 and Table 2. The % ID shows the percentage of the accumulated dose in each organ. Because the weights of the organs are quite different, % ID/g is more useful for evaluating the organ accumulation. The level of 5 nm ^211^At-AuNPs@mPEG accumulated in tumor tissue at 3 h post-administration was 2.25 ± 0.67 % ID/g, which was higher than that of 30 nm ^211^At-AuNPs@mPEG (1.29 ± 0.17 % ID/g). After 24 h, the accumulation of AuNPs could still be observed in the tumor tissues, which were 1.80 ± 0.20 % ID/g and 0.85 ± 0.21% ID/g for 5 nm and 30 nm ^211^At-AuNPs@mPEG, respectively. The data obtained from urine indicated rapid clearance between 3 and 24 h post-administration.

The biodistribution (% ID/g and % ID) of 5 nm ^211^At-AuNPs@H16 and ^211^At-AuNPs@H16/RGD is shown in Table 3 and Table 4. At 3 h after the injection, the accumulation of ^211^At-AuNPs@H16 and ^211^At-AuNPs@H16/RGD was high in the liver (Table 2 and Table 4), i.e., 17.83 ± 8.54 % ID/g and 16.02 ± 7.08 % ID/g, respectively. A higher accumulation of RGD peptide-modified AuNPs (41.59 ± 2.20 % ID/g) in the liver 24 h post-administration was observed. However, in the tumor tissues, 1.36 ± 0.44 % ID/g and 2.34 ± 0.94 % ID/g of 5 nm ^211^At-AuNPs@H16 and 5 nm ^211^At-AuNPs@H16/RGD, respectively, were accumulated at 3 h post-administration. After 24 h, 1.31 ± 0.27 % ID/g of ^211^At-AuNPs@H16 and 2.07 ± 0.47 % ID/g of ^211^At-AuNPs@H16/RGD remained in the tumor tissue. Compared to mPEG-modified AuNPs, tumor enrichment of AuNPs was not significantly improved by peptide modification.

### 3.3. In Vivo Therapeutic Effect

Considering the higher accumulation of ^211^At-AuNPs@H16 and ^211^At-AuNPs@H16/RGD in the liver, we assume these have a stronger side effect and that they may not be suitable for intravenous administration. Since 5 nm ^211^At-AuNPs@mPEG showed relatively higher accumulation in tumor tissues and relatively lower accumulation in other organs, we ultimately used 5 nm ^211^At-AuNPs@mPEG to investigate the in vivo therapeutic effect. 

**Figure 2:**
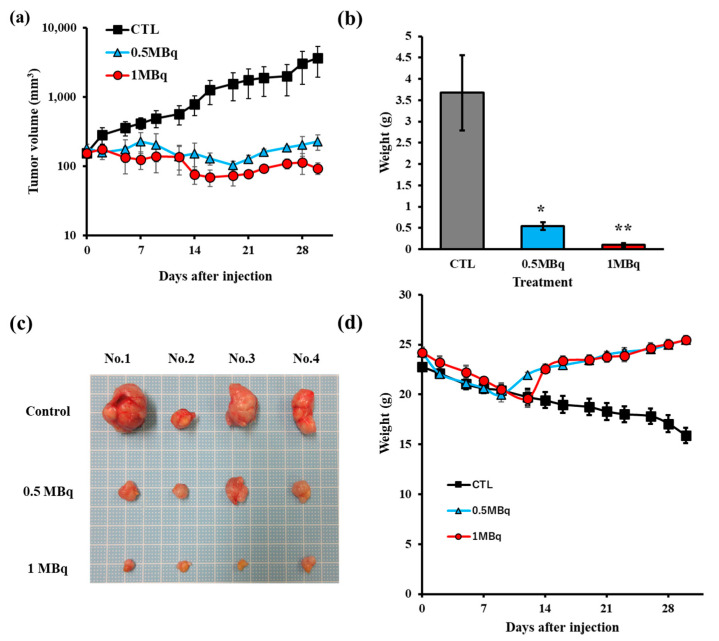
Tumor growth inhibition by 5 nm ^211^At-AuNPs@mPEG in the PANC-1 xenograft model. At 2 weeks following tumor inoculation, 5 nm ^211^At-AuNPs@mPEG (1 MBq or 0.5MBq/mouse) was injected, and 5 nm AuNPs@mPEG was injected to the control group (CTL). Tumor size (**a**) and weight (**d**) were measured thrice per week. Tumors were enucleated and weighed 30 days after injection (**b**,**c**). (* *p* < 0.05, ** *p* < 0.01).

After administration, no inflammation or abnormalities were observed around the injection site. The tumor size change after administration of 5 nm ^211^At-AuNPs@mPEG (1 MBq/mL or 0.5 MBq/mL) and AuNPs@mPEG (0 MBq/mL) are shown in Figure 2a. After one month, the mice were euthanized, and the tumors were removed to measure their weight, as shown in Figure 2b,c. Tumor proliferation was significantly inhibited by treatment with ^211^At-AuNPs@mPEG in the 1 MBq and 0.5 MBq groups. Although % ID in the tumors was 2.03 ± 0.27 % ID (3 h) and 1.92 ± 0.77 % ID (24 h), the tumor growth was suppressed by treatment with ^211^At-AuNPs. The mouse body weight slightly decreased after the administration of ^211^At-AuNPs@mPEG, but recovered after 2 weeks, as shown in Figure 2d.

### 3.4. Cellular Uptake of AuNPs and DNA DSBs In Vitro

The cellular uptake of AuNPs without ^211^At labeling was investigated using reflectance imaging. Cold AuNPs were added to the cell and incubated for 24 h. As shown in Figure S3A, 5 nm AuNPs@H16 and AuNPs@H16/RGD were accumulated in the cells, while the uptake of 5 nm and 30 nm mPEG-modified AuNPs was quite low in vitro. 

The internalization of 120 nm AuNPs@mPEG in the breast cancer (MDA-MB-231) and normal mammary gland tissue (Hs 578Bst) cell lines were compared (Figure S5). Many visible black AuNP aggregates were observed in the MDA-MB-231 cells, as shown in DIC (differential interference contrast) images (Figure S5), whereas in the Hs 578Bst cell line, the AuNPs uptake was low and AuNPs were evenly distributed within the cells. In addition, the cellular uptake of ^211^At-AuNPs was quantified by determining the radioactivity of PANC-1 cell lysates as shown in Figure S3B. After 5 h of incubating the cells with ^211^At-AuNPs, the internalization level of the two kinds of mPEG-modified AuNPs was low, whereas the internalization of the two kinds of peptide-modified AuNPs was significantly higher than that of mPEG-modified AuNPs. 

γH2AX was used as the marker of DNA DSB induction. The results of the DSB induction are shown in Figure S4. ^211^At-AuNPs@mPEG (5 nm and 30 nm) induced low levels of DSB. The two kinds of peptide-modified AuNPs caused a higher DNA DSB induction after 5 h of incubation with 1 MBq/mL of ^211^At-AuNPs@H16 or ^211^At-AuNPs@H16/RGD in a concentration-dependent manner.

## 4. Discussion

Gold nanoparticles have been used as a delivery carrier for ^211^At due to the strong affinity of astatine to gold [13,18,19,25,26]. We recently reported that ^211^At-AuNPs@mPEG exhibited cytotoxicity when they were absorbed by the tumor cells and the intratumoral administration of ^211^At-AuNPs@mPEG strongly suppressed the cancer growth in a particle size-dependent manner. Smaller particles showed a more rapid distribution in tumor regions, and 5 nm ^211^At-AuNPs@mPEG showed the highest suppression of cancer growth among various sizes of AuNPs tested via intratumoral administration [13]. A similar nanoseed brachytherapy using ^211^At-labeled gold nanostars has also reported significant inhibition of the growth of human gliomas in a murine model [25].

Here, we demonstrated that 5 nm ^211^At-AuNPs@mPEG significantly suppressed tumor growth in a pancreatic cancer model via intravenous administration. The accumulation of ^211^At-AuNPs@mPEG in PANC-1 tumors occurred via passive targeting through the EPR effect. The 5 nm ^211^At-AuNPs@mPEG showed a higher % ID of accumulation in tumors than 30 nm ^211^At-AuNPs@mPEG, suggesting the higher vascular permeability and the higher diffusion into tumor tissue. Many factors affect the EPR effect of AuNPs, including the size, surface modification of nanoparticles, and tumor microenvironment [27,28,29,30,31]. Sykes et al. reported that passively targeted AuNPs with diameters less than 45 nm (core size, hydrodynamic diameter 78 nm) easily permeate the tumor [32]. Liu et al. reported that 30 nm gold nanostars have higher tumor uptake and deeper penetration into the tumor interstitial space than their 60 nm counterparts [33]. The % ID observed in the thyroid and stomach is likely due to the accumulation of ^211^At-AuNPs, but not free ^211^At, since ^211^At-AuNP was proven to be stable in the previous studies [13,19]. The present % ID levels in thyroid and stomach were much smaller than the levels of free ^211^At [19]. AuNPs have been reported to be widely distributed in various organs such as blood, liver, lungs, spleen, kidneys, brain, heart, and stomach in addition to the excretion to feces and urine. Smaller gold nanoparticles tend to be more easily distributed in each organ. [21,34,35,36,37]. The radioactivity detected in urine indicated that ^211^At-AuNPs were excreted via the kidneys and 5 nm AuNPs were more readily excreted than 30 nm ones. Though distribution in feces, muscle, heart, etc., was not measured, the trends in biodistribution in various organs are consistent with the previous studies.

In contrast, the in vitro cellular uptake and DNA DSB induction of both mPEG-modified AuNPs were low. We also report that in vitro uptake of ^211^At-labeled AuNPs does not correlate with their in vivo anticancer effects in our previous intratumoral administration study [13]. The 120 nm ^211^At-AuNPs@mPEG showed evident cytotoxicity in an in vitro cellular assay, while ^211^At-labeled AuNPs of smaller sizes did not show cytotoxicity. The 120 nm AuNPs were precipitated by gravity, allowing the AuNPs to contact cells, and the uptake of the AuNPs into the cells led to cell death. The other AuNPs were dispersed in solution and were not taken up by the cells in the in vitro system, therefore no cytotoxicity was observed. In contrast, the intratumoral administration of 5 nm ^211^At-AuNPs@mPEG exhibited the most potent anticancer activity in our previous study [13]. These results indicate that 5 nm AuNPs are efficiently dispersed in tumor tissue and rapidly internalized into cancer cells in vivo by both intravenous and intratumoral administrations.

Significant adverse effects were not observed following the intravenous administration of 5 nm ^211^At-AuNPs@mPEG, although a slight weight loss was observed in the early phase. The difference in uptake of AuNPs between cancer cells and normal cells can explain the low toxicity of 5 nm ^211^At-AuNPs@mPEG to normal cells. The “eating” ability of cancer cells should be much higher than that of normal cells, since tumors require abundant nutrients to grow. We thus compared the internalization of AuNPs@mPEG in both breast cancer and normal mammary gland tissue cell lines. The 120 nm AuNPs@mPEG was used for the in vitro uptake owing to the reasons that we previously explained [13]. As expected, the quantity of uptake and subsequent distribution in tumor cells were different from those in the normal cells (Figure S5). Prominent black spots showed in the DIC images representing the aggregates of AuNPs, were present in cancer cells. Liu et al. reported that non-phagocytic HepG2 cells took up positively charged AuNPs, but not as many negatively-charged AuNPs, while phagocytic RAW264.7 cells efficiently took up both negatively- and positively-charged AuNPs [38]. Since the zeta-potential of 5 nm AuNPs@mPEG was −14.9 mV (Table S1), there is no contradiction with this report.

Considering the short half-life of ^211^At, the difference in the uptake rate of AuNPs should significantly impact on the selective killing of tumors by ^211^At. The use of ^211^At-labeled AuNPs allows the spatiotemporal selective uptake of radionuclides into tumor cells due to the high uptake capacity of the nanoparticles into tumor cells and passive targeting through the EPR effect.

We also investigated the gold nanoparticles (5 nm AuNPs@H16 and AuNPs@H16/RGD) that were modified with oligohistidine peptide [20] and cyclic RGD [21,22], which can target tumor pH and the integrin α_v_β_3_, respectively, to promote their tumor-targeting ability and cellular uptake. RGD peptide, which can bind with the tumor marker integrin α_v_β_3_, is widely used for targeted delivery [21,22]. Oligohistidine peptides can be effectively internalized into cells, and can be used for tumor-targeted approaches due to their pH-responsiveness [20]. The in vitro study showed significant internalization and higher DSB induction of the two kinds peptide-modified AuNPs. 

However, the accumulation of both AuNPs@H16 and AuNPs@H16/RGD in tumors was not high, but significantly higher in the liver compared with mPEG-modified AuNPs. Due to the high accumulation in the liver, these AuNPs are not applicable for treatments via intravenous administration. Considering the high internalization and significant DSB induction ability of the peptide-modified AuNPs, these are expected to be beneficial for treatments via local administration. In fact, Dziawer et al. reported that trastuzumab-modified ^211^At-labeled AuNPs, which exhibited high affinity and cytotoxicity to the HER2-overexpressing human ovarian SKOV-3 cell line [26], is a potential prospective tool for the local treatment of HER2-positive breast cancer.

In fact, the shape, surface modification, and charge of AuNPs have a significant impact on their toxicity, stability, and pharmacokinetics. The appropriate modification of the AuNP surface may solve these issues. For example, Lee et al. revealed that the native cytotoxicity of AuNPs is regulated by the direction or the magnitude of the surface charge as well as the spatial arrangement of a hydrophobic moiety neighboring the positive charge [39]. 

The surface modification of AuNPs is also important to improve tumor selectivity [40,41,42]. For example, glucose-modified AuNPs were developed to target cancer cells [43,44]. AuNPs bearing methoxypolyethylene glycol-graft-poly(L-lysine) copolymer (MPEG-gPLL) exhibited high uptake and very low toxicity in human endothelial cells, but showed a high dose-dependent toxicity in epithelioid cancer cells [45]. Surface-modified AuNPs with chiral polymers (poly(acryloyl-L, D and racemic valine)) induced cytotoxicity towards MDA-MB-231 cells mainly through autophagy. Polyacryloyl-D-valine AuNPs exhibited high autophagy-inducing activity and suppressed the tumor growth by the intratumoral injection [46]. AuNPs loaded with technetium-99m and methotrexate (^99m^Tc-Mex) were developed for tumor diagnosis and treatment [47]. Methionine-modified ^99m^Tc AuNPs (TMGN) were also developed for SPECT tumor imaging [48]. TMGN showed nearly three-fold higher tumor accumulation (3.9 ± 0.35 % ID/g) and higher tumor retention than ^99m^Tc AuNPs (TM) in a mouse model. Modification with multiple targeting ligands to the nanoparticles is expected to further improve targeting efficiency due to the cooperative effect. For example, the cellular uptake of nanographene oxide (nGO) modified with RGD and folic acid (FA) was increased compared to RGD-nGO or FA-nGO [49]. This method can be readily applied to AuNPs for better tumor targeting.

As described above, the in vivo efficiency was unable to be predicted from the in vitro results. In this study, we used monolayer cell cultures for the evaluation of biological efficacy, which are typically used as in vitro testing platforms. Since the AuNPs without cell adhesion molecules are suspended in solution and are difficult to make contact with cell surfaces in vitro, it is difficult to estimate the cellular uptake capacity in vitro. Goodman et al. indicated that in vitro evaluation using monolayer cell cultures sometimes poorly predicts the efficacy of the nano drugs in vivo that may be attributed in part to the inability of two-dimensional cultures to reproduce the tumor microenvironment [50]. It is better to use 3D tissue culture systems to evaluate the nanoparticles in vitro in order to predict in vivo results more accurately.

From a synthetic point of view, the simple operation and short-time synthesis of ^211^At-AuNPs are major advantages in the synthetic process. The ^211^At-labeled AuNPs can be easily obtained by simply mixing AuNPs and ^211^At solution, although the ^211^At solution was found to be a mixture of chemical species probably with different valences, such as At^+^, At^−^, etc., in the previous study [9]. Various surface-modified ^211^At-labeled AuNPs were also readily obtained at high RCY. In addition, the At–Au bond was reported to be stable both in theory and experimentally [13,18,19,25,26]. Another major problem was that ^211^At or ^211^At-labeled compounds readily stick to the reaction vessel and reduce the yields of labeled products. Nevertheless, the ^211^At adsorption capacity to AuNPs is stronger than that to the reaction vessel, and the adsorption amount of ^211^At in reaction tubes was quite low. In addition, because of high RCY, no purification was necessary which is useful when using short-lived isotopes.

Taking the above advantages into consideration, ^211^At-AuNPs seems to be very suitable for cancer treatment development and has potential clinical applications in the future.

## 5. Conclusions

This study sheds new light on the substantial potential of AuNPs as a carrier for the targeted delivery of ^211^At via intravenous administration. Our results showed that AuNP is a satisfactory carrier for ^211^At delivery, due to the simple and efficient synthesis process and high stability. The intravenous administration of 5 nm ^211^At-AuNPs@mPEG has a significant anti-tumor effect. We took advantage of the EPR effect and the fact that cancer cells have a higher nanoparticle uptake rate than normal cells, and successfully developed a novel TAT compound.

## Figures and Tables

**Table 1:** Comparison of percentage distribution of injected dose per gram (% ID/g) of 5 nm and 30 nm ^211^At-AuNPs@mPEG.

	3 h		24 h	
	5 nm ^211^At-AuNPs@mPEG	30 nm ^211^At-AuNPs@mPEG	5 nm ^211^At-AuNPs@mPEG	30 nm ^211^At-AuNPs@mPEG
Thyroid	12.12 ± 5.69	2.61 ± 0.39	4.560 ± 1.05	6.98 ± 2.33
Liver	2.88 ± 0.82	5.87 ± 3.79	2.18 ± 0.37	2.08 ± 1.16
Stomach	11.73 ± 5.22	2.03 ± 0.56	2.45 ± 0.60	4.71 ± 2.19
Small intestine	2.10 ± 0.76	0.85 ± 0.13	0.81 ± 0.09	0.74 ± 0.23
Colon	1.36 ± 0.41 ^#^	0.34 ± 0.02	0.88 ± 0.18	0.67 ± 0.24
Kidney	2.80 ± 0.58 ^#^	1.27 ± 0.21	1.90 ± 0.26	1.17 ± 0.38
Blood	7.06 ± 0.88 *^###^	0.59 ± 0.04	2.60 ± 0.54 ^#^	0.57 ± 0.20
Urine	8.99 ± 3.23	3.73 ± 1.67	14.68 ± 1.97 ^#^	14.90 ± 3.72
Tumor	2.25 ± 0.67	1.29 ± 0.17	1.80 ± 0.20 ^#^	0.85 ± 0.21

* Mean ± standard error (SE) of % ID/g is shown in the table. Significant difference between 3 and 24 h is indicated as * *p* < 0.05, and between 5 nm and 30 nm ^211^At-AuNPs@mPEG at 24 h as ^#^ *p* < 0.05, ^###^ *p* < 0.001.

**Table 2:** Comparison of percentage distribution of injected dose (% ID) of 5 nm and 30 nm ^211^At-AuNPs@mPEG.

	3 h		24 h	
	5 nm ^211^At-AuNPs@mPEG	30 nm ^211^At-AuNPs@mPEG	5 nm ^211^At-AuNPs@mPEG	30 nm ^211^At-AuNPs@mPEG
Thyroid	0.67 ± 0.26	0.19 ± 0.02	0.31 ± 0.05	0.42 ± 0.12
Liver	3.97 ± 1.17	7.61 ± 5.22	2.68 ± 0.41	2.70 ± 1.42
Stomach	3.96 ± 2.00	0.52 ± 0.17	0.64 ± 0.19	1.23 ± 0.51
Small intestine	1.93 ± 0.73	0.82 ± 0.12	0.86 ± 0.10	0.81 ± 0.18
Colon	0.70 ± 0.39	0.08 ± 0.02	0.23 ± 0.06	0.14 ± 0.03
Kidney	1.23 ± 0.25 ^#^	0.55 ± 0.08	0.81 ± 0.10	0.48 ± 0.14
Blood	1.79 ± 0.43 ^#^	0.30 ± 0.09	1.26 ± 0.20	0.29 ± 0.07 ^##^
Urine	0.57 ± 0.22	0.63 ± 0.36	2.24 ± 0.58 *	0.14 ± 0.11 ^#^
Tumor	2.03 ± 0.62	1.45 ± 0.21	1.92 ± 0.77	0.91 ± 0.44

* Mean ± standard error (SE) of % ID is shown in the table. Significantly difference between 3 and 24 h is indicated as * *p* < 0.05, and between 5 nm and 30 nm ^211^At-AuNPs@mPEG in 24 h as ^#^ *p* < 0.05 and ^##^ *p* < 0.01.

**Table 3:** Comparison of percentage distribution of injected dose per gram (% ID/g) of ^211^At-AuNPs by peptide modification.

	3 h		24 h	
	5 nm ^211^At-AuNPs@H16	5 nm ^211^At-AuNPs@H16/RGD	5 nm ^211^At-AuNPs@H16	5 nm ^211^At-AuNPs@H16/RGD
Thyroid	3.64 ± 1.38	3.29 ± 1.29	3.34 ± 0.47	2.60 ± 0.51
Liver	17.83 ± 8.54	16.02 ± 7.08 *	16.91 ± 6.48	41.59 ± 2.20 ^#^
Stomach	3.74 ± 1.19	3.89 ± 1.23	1.73 ± 0.20	2.45 ± 0.56
Small intestine	0.85 ± 0.28	1.11 ± 0.31	0.46 ± 0.06	1.01 ± 0.07 ^###^
Colon	0.68 ± 0.19	0.67 ± 0.15	0.43 ± 0.11	0.72 ± 0.10
Kidney	1.74 ± 0.46	1.64 ± 0.56	0.85 ± 0.12	2.09 ± 0.28 ^###^
Blood	6.91 ± 1.48 **	7.44 ± 1.59 **	0.64 ± 0.11	0.44 ± 0.05
Urine	12.98 ± 5.97	8.04 ± 1.79 **	4.17 ± 1.29	0.44 ± 0.43 ^#^
Tumor	1.36 ± 0.44	2.34 ± 0.94	1.31 ± 0.27	2.07 ± 0.47

* Mean ± standard error (SE) of % ID/g is shown in the table. Significantly difference between 3 and 24 h is indicated as * *p* < 0.05 and ** *p* < 0.01, and between 5 nm ^211^At-AuNPs@H16 and 5 nm ^211^At-AuNPs@H16/RGD in 24 h as ^#^ *p* < 0.05, and ^###^ *p* < 0.001.

**Table 4:** Comparison of percentage distribution of injected dose (% ID) of 5 nm and 30 nm ^211^At-AuNPs@mPEG.

	3 h		24 h	
	5 nm ^211^At-AuNPs@H16	5 nm ^211^At-AuNPs@H16/RGD	5 nm ^211^At-AuNPs@H16	5 nm ^211^At-AuNPs@H16/RGD
Thyroid	0.23 ± 0.09	0.17 ± 0.06	0.17 ± 0.02	0.15 ± 0.05
Liver	23.32 ± 10.85	20.73 ± 8.52	22.09 ± 7.91	44.26 ± 1.78 *^#^
Stomach	1.45 ± 0.41	1.54 ± 0.46	0.66 ± 0.10	0.83 ± 0.15
Small intestine	1.18 ± 0.39	1.34 ± 0.39	0.53 ± 0.08	1.08 ± 0.11 ^##^
Colon	0.13 ± 0.04	0.19 ± 0.07	0.14 ± 0.06	0.17 ± 0.01
Kidney	0.69 ± 0.18	0.67 ± 0.26	0.34 ± 0.04	0.69 ± 0.10 ^#^
Blood	2.91 ± 0.41	3.79 ± 0.64	0.25 ± 0.03 ***	0.23 ± 0.03 **
Urine	1.27 ± 0.85	0.42 ± 0.16	0.09 ± 0.03	0.53 ± 0.17 ^#^
Tumor	0.28 ± 0.14	0.45 ± 0.19	0.23 ± 0.09	0.15 ± 0.03

* Mean ± standard error (SE) of % ID is shown in the table. Significantly difference between 3 and 24 h is indicated as * *p* < 0.05, ** *p* < 0.01, *** *p* < 0.001, and between 5 nm ^211^At-AuNPs@H16 and 5 nm ^211^At-AuNPs@H16/RGD in 24 h ^#^ *p* < 0.05, ^##^ *p* < 0.01.

## Data Availability

Data available on request.

## Supplementary Materials

The following supporting information can be downloaded at: https://www.mdpi.com/article/10.3390/pharmaceutics14122705/s1, Figure S1: Transmission electron microscopy (TEM) images of four AuNPs; Figure S2: Evaluation of ^211^At-labeling reaction; Figure S3: Evaluation of AuNPs internalization; Figure S4: DNA double-strand break induced by ^211^At-AuNPs; Figure S5: Comparation of AuNPs internalization in cancer cells and normal cells; Table S1: Hydrodynamic diameter, polydispersity index, and zeta-potential of AuNPs; Table S2: Stability evaluation of two kinds of peptides modified AuNPs in PB; Table S3: Evaluation condition of ^211^At labeling.
