# Supplementary material for: Astatine-211-Labeled Gold Nanoparticles for Targeted Alpha-Particle Therapy via Intravenous Injection
Source: Pharmaceutics. 2022 Dec 2;14(12):2705. doi: 10.3390/pharmaceutics14122705 (PMC9782038; doi:10.3390/pharmaceutics14122705)
Supplement: Supplementary file 1 [file pharmaceutics-14-02705-s001.zip › pharmaceutics-1996863-supplementary.pdf]

Supplementary Information

Astatine-211-Labeled Gold Nanoparticles for Targeted Alpha-particle Therapy via Intravenous Injection

Xuhao Huang [1], Kazuko Kaneda-Nakashima [2,3], Yuichiro Kadonaga [4], Kazuya Kabayama [1,2,3], Atsushi Shimoyama [1,2,3], Kazuhiro Ooe [2,3], Hiroki Kato [4], Atsushi Toyoshima [2,3], Atsushi Shinohara [2,5], Hiromitsu Haba [6], Yang Wang [6] and Koichi Fukase[1,2,3,*]

1. Department of Chemistry, Graduate School of Science, Osaka University, 1-1 Machikaneyama, Toyonaka 560-0043, Osaka, Japan

2. Division of Science, Institute for Radiation Sciences, Osaka University, 1-1 Machikaneyama, Toyonaka 560-0043, Osaka, Japan

3. Core for Medicine and Science Collaborative Research and Education, Forefront Research Center, Graduate School of Science, Osaka University, 1-1 Machikaneyama, Toyonaka 560-0043, Osaka, Japan

4. Department of Nuclear Medicine and Tracer Kinetics, Osaka University Graduate School of Medicine, 2-2 Yamadaoka, Suita 565-0871, Osaka, Japan

5. Faculty of Health Science, Osaka Aoyama University, 2-11-1 Niina, Minoh 562-8580, Osaka, Japan

6. Nishina Center for Accelerator-Based Science, RIKEN, 2-1 Hirosawa, Wako 351-0198, Saitama, Japan

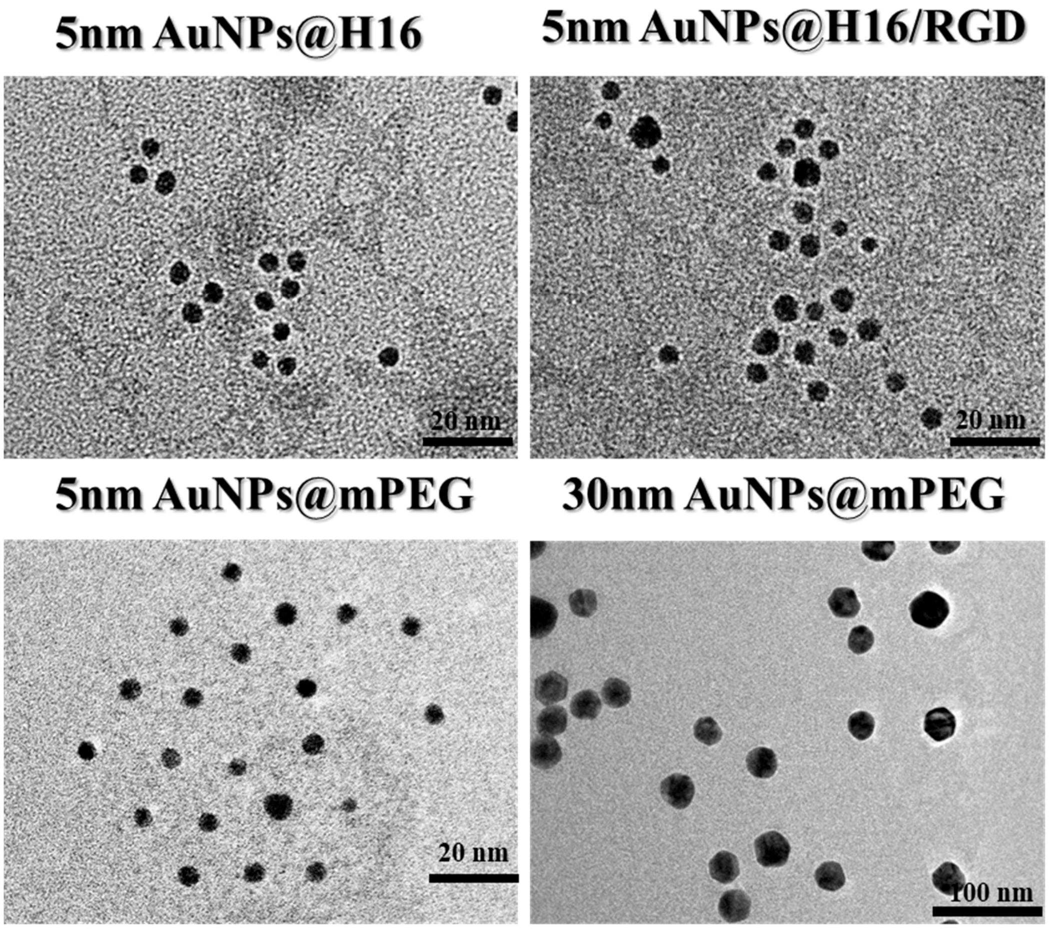

Figure S1. Transmission electron microscopy (TEM) images of four AuNPs.

Table S1. Hydrodynamic diameter, polydispersity index and zeta-potential of AuNPs.

	Hydrodynamic diameter (Average)	Polydispersity Index	Zeta-potential
5 nm AuNPs (Citrate buffer)	18.58 nm	0.2362	-25.8 mV
30 nm AuNPs (Citrate buffer)	33.59 nm	0.2356	-41.0 mV
5 nm AuNPs@mPEG (Water)	39.41 nm	0.1245	-14.9 mV
30 nm AuNPs@mPEG (Water)	57.58 nm	0.2998	-20.8 mV
5 nm AuNPs@H16 (PB pH6.0)	28.96 nm	0.3353	-0.5 mV
5 nm AuNPs@H16/RGD (PB pH6.0)	42.64 nm	0.4037	-1.2 mV

Table S2. Stability evaluation of two kinds of peptides modified AuNPs in PB.

	Hydrodynamic diameter	Polydispersity Index
5 nm AuNPs@H16 (Diluted to PB pH7.4)	32.45 nm	0.3962
5 nm AuNPs@H16 (Diluted to PB pH8.0)	43.69 nm	0.3606
5 nm AuNPs@H16/RGD (Diluted to PB pH7.4)	43.17 nm	0.3938
5 nm AuNPs@H16/RGD (Diluted to PB pH8.0)	42.15 nm	0.5424

Table S3. Evaluation condition of ^{211}At labeling.

Samples	OD	Concentration particles/mL	AuNPs solution volume	^{211}At solution (MBq/30 μL)	Radio-chemical yield
5 nm AuNPs@mPEG	1	5.5×10^{13}	70 μL	4.7 ~ 5.6	99.5 %
30 nm AuNPs@mPEG	1	1.8×10^{11}	70 μL	4.7 ~ 5.6	97.5 %
5 nm AuNPs@H16	1	5.5×10^{13}	70 μL	4.7 ~ 5.6	91.9 %
5 nm AuNPs@H16/RGD	1	5.5×10^{13}	70 μL	4.7 ~ 5.6	93.4 %

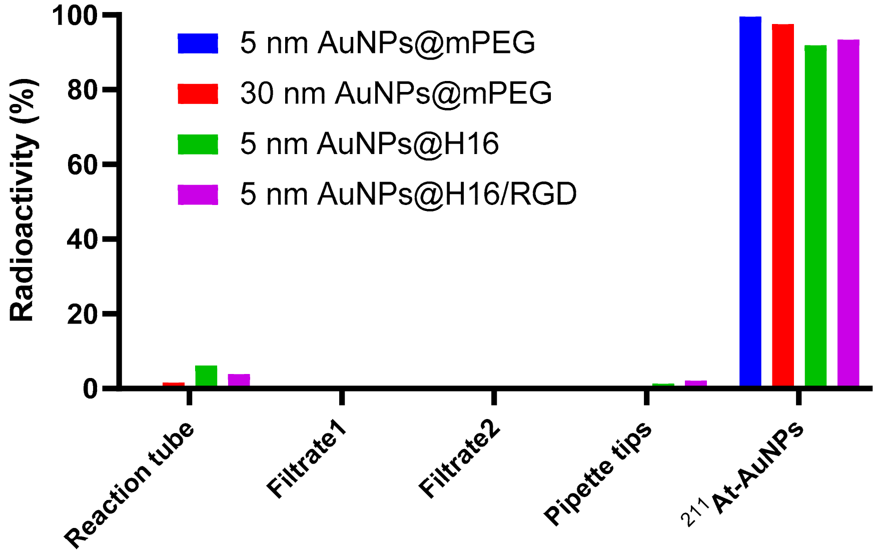

Figure S2. Evaluation of ^{211}At labelling reaction.

^{211}At solution was mixed with AuNPs solution for five minutes, then the mixture was centrifuged for twice. The radioactivity of reaction tube, filtrate 1, filtrate 2, pipette tips and ^{211}At-AuNPs was measured in order to calculated the radiochemistry yield.

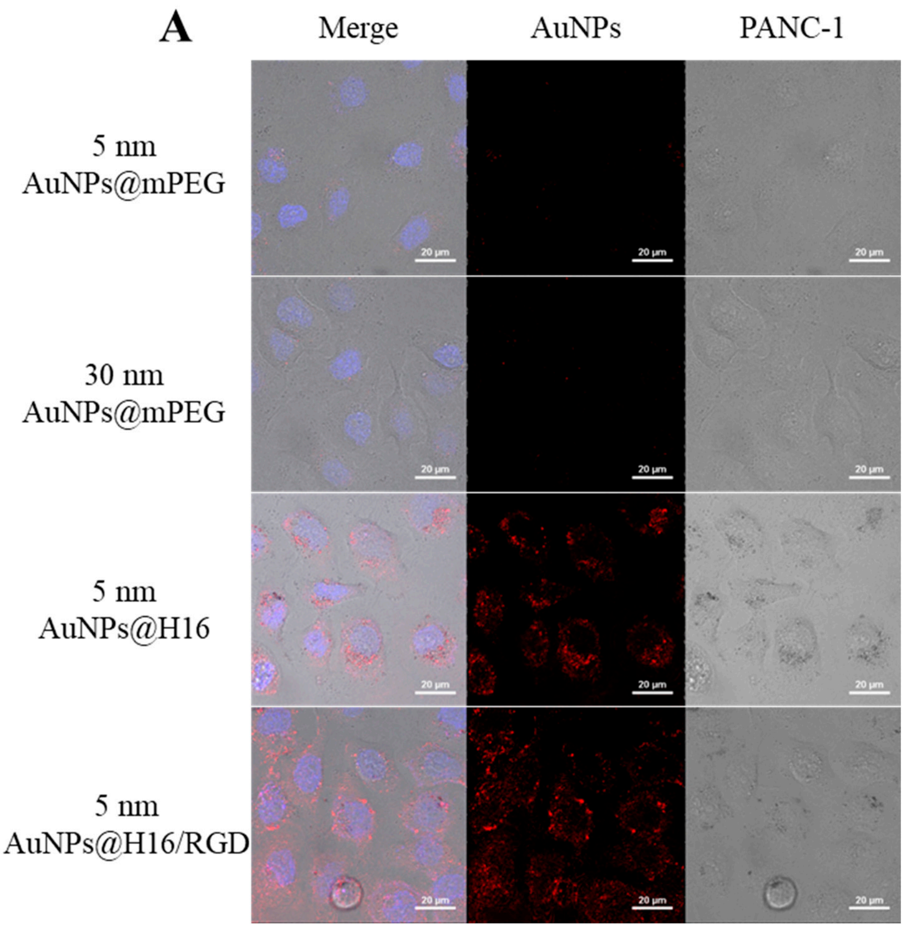
A
Merge
AuNPs
PANC-1
5 nm
AuNPs@mPEG
30 nm
AuNPs@mPEG
5 nm
AuNPs@H16
5 nm
AuNPs@H16/RGD
20 μm

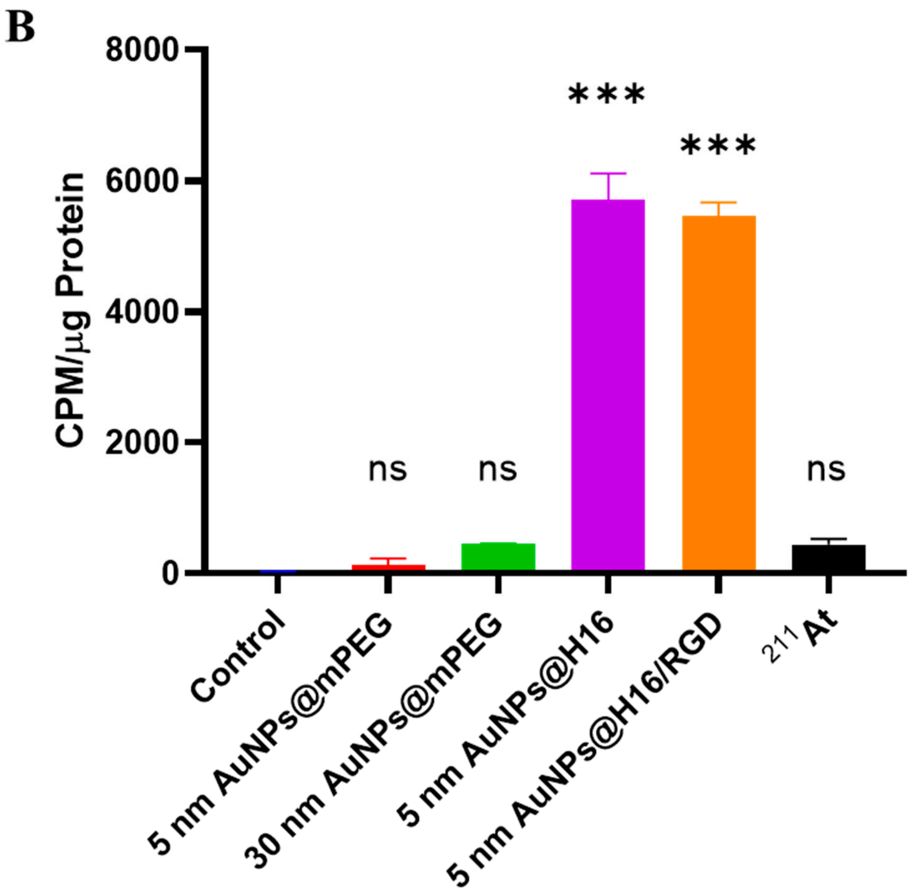

Figure S3. Evaluation of AuNPs internalization. (A) Imaging of AuNPs' internalization in PANC-1 cell line. AuNPs were add to the PANC-1 cells and incubated for 24 hours, then reflectance imaging was performed. Red is AuNPs, blue is cell nucleus. Bar = 20μm. (B) Internalization quantification of ^{211}At-AuNPs in PANC-1 cell line. ^{211}At-AuNPs were add to the PANC1 cells and incubated for 5 hours, then the radioactivity/μg protein was measured. (***$p < 0.001$).

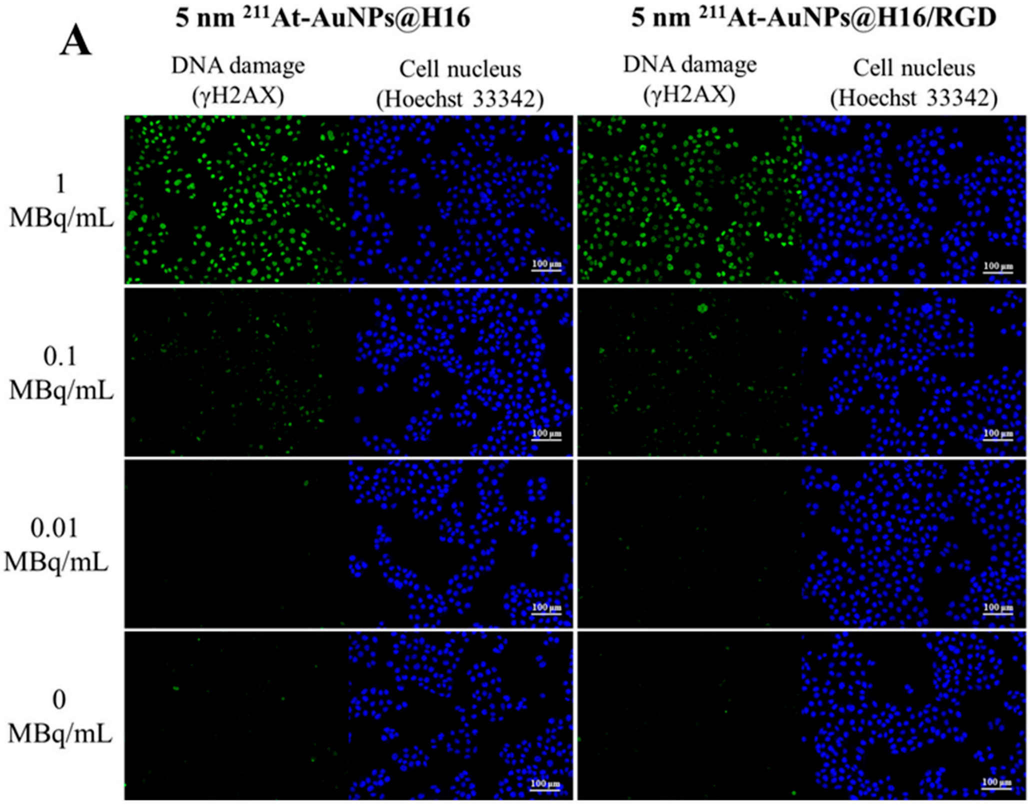

A
5 nm ^{211}At-AuNPs@H16
5 nm ^{211}At-AuNPs@H16/RGD
DNA damage (γH2AX)
Cell nucleus (Hoechst 33342)
DNA damage (γH2AX)
Cell nucleus (Hoechst 33342)
1 MBq/mL
0.1 MBq/mL
0.01 MBq/mL
0 MBq/mL

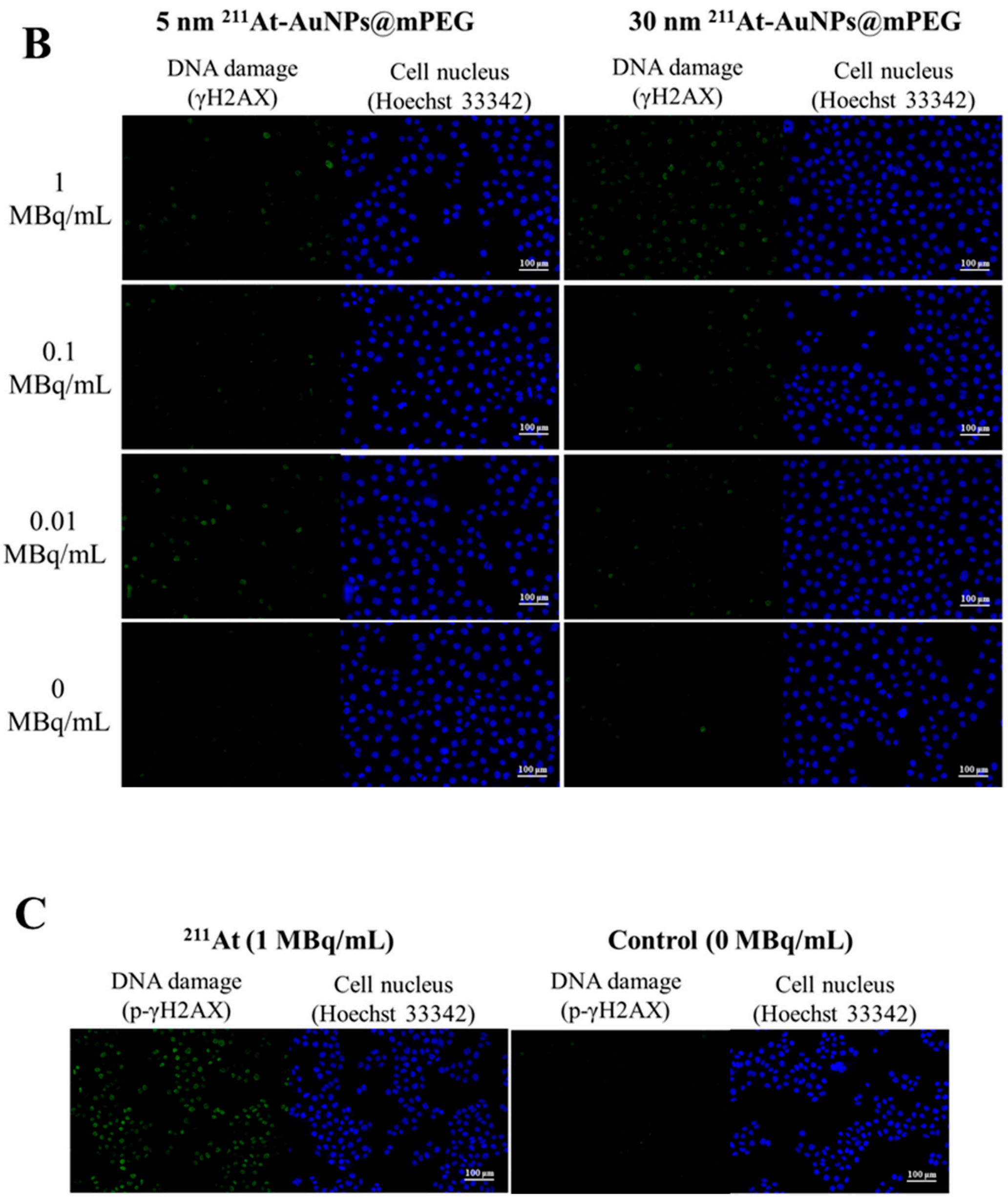
B
5 nm 211At-AuNPs@mPEG
30 nm 211At-AuNPs@mPEG
DNA damage (γH2AX)
Cell nucleus (Hoechst 33342)
DNA damage (γH2AX)
Cell nucleus (Hoechst 33342)
1 MBq/mL
0.1 MBq/mL
0.01 MBq/mL
0 MBq/mL
C
211At (1 MBq/mL)
Control (0 MBq/mL)
DNA damage (p-γH2AX)
Cell nucleus (Hoechst 33342)
DNA damage (p-γH2AX)
Cell nucleus (Hoechst 33342)

D

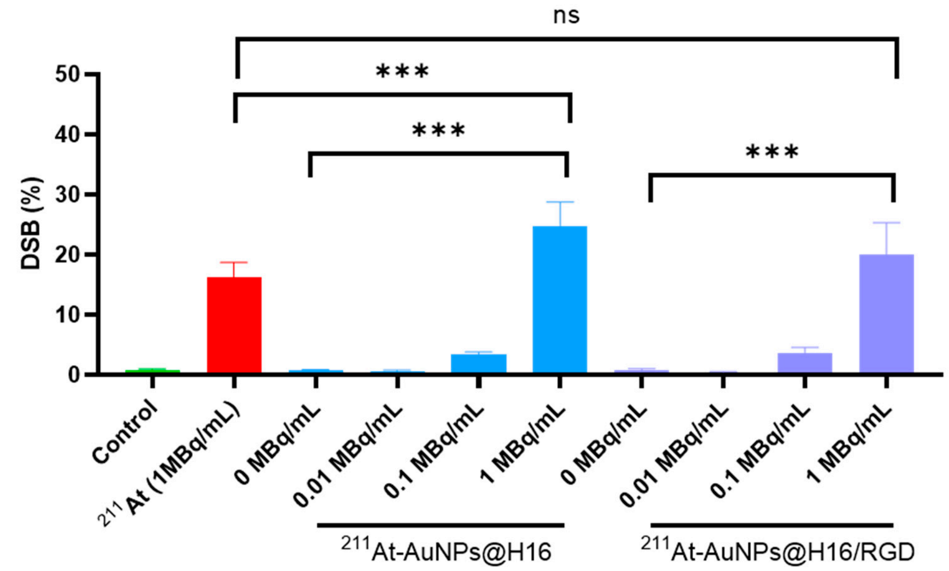

E

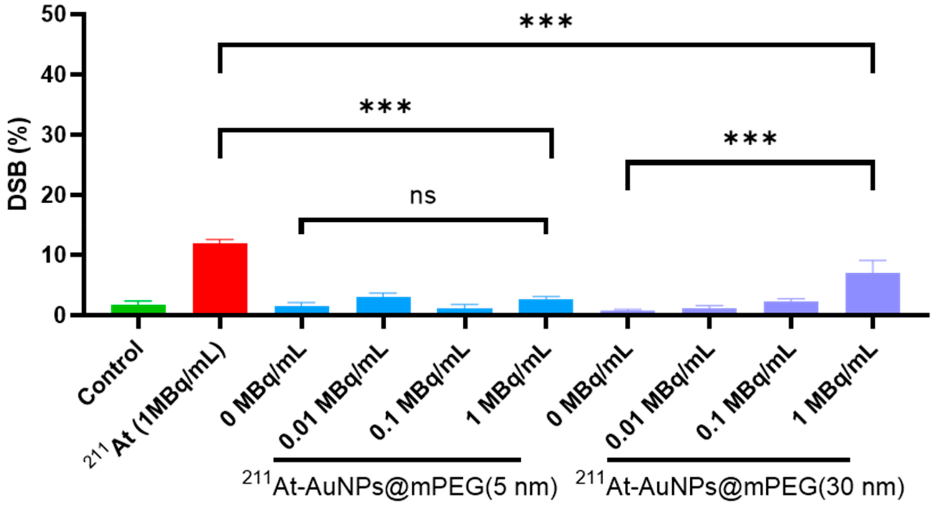

Figure S4. DNA double-strand break induced by ^{211}At-AuNPs. (A–C) Representative images of immunofluorescence staining; green (γH2AX), blue (cell nuclei). (A) DSB induced by 5 nm ^{211}At-AuNPs@H16 and ^{211}At-AuNPs@H16/RGD; (B) DSB induced by 5 nm and 30 nm ^{211}At-AuNPs@mPEG; (C) DSB induced by free ^{211}At and PBS (Control), (D,E) Quantitative analysis of the DSB induction. DSB% = Average fluorescence intensity of γH2AX / Average fluorescence intensity cell nuclei × %. (ns: no significance, ***$p < 0.001$).

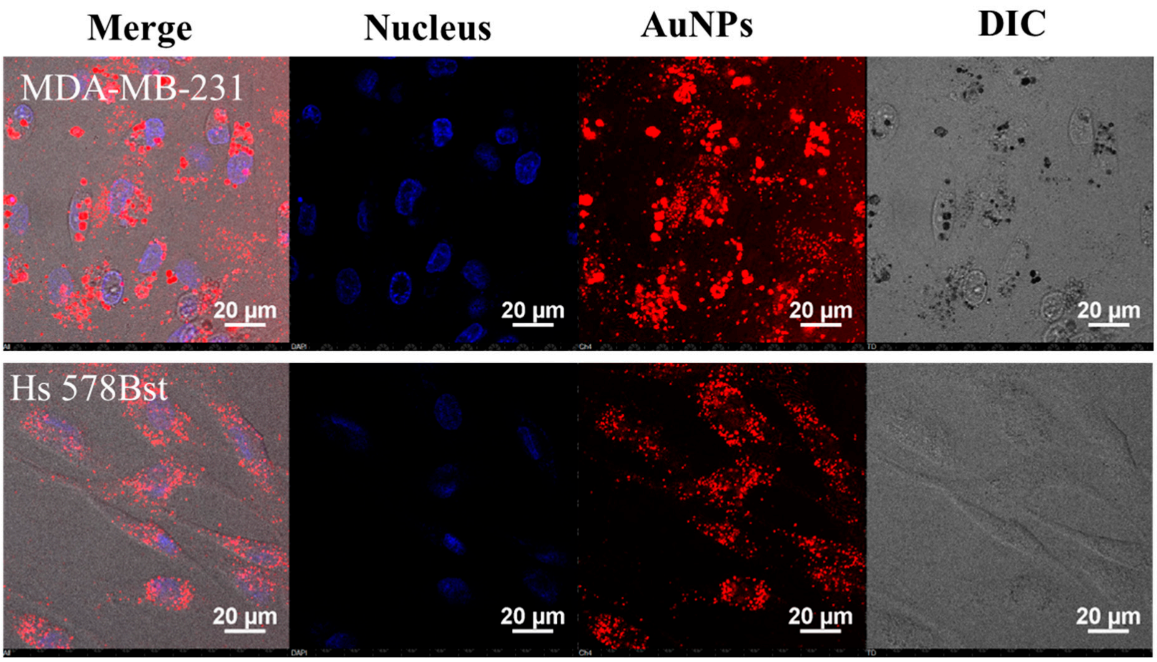

Figure S5. Comparation of AuNPs internalization in cancer cells and normal cells.

Imaging of 120 nm AuNPs@mPEG internalization in MDA-MB-231 and Hs 578Bst cell lines. AuNPs were add to the PANC-1 cells and incubated for 24 hours, then reflectance imaging was performed. Red is AuNPs, blue is cell nucleus, DIC is differential interference contrast. Bar = 20μm.
